# Structure of Full-Length Src Kinase and Its Key Phosphorylated States: Molecular Dynamics Study

**DOI:** 10.3390/ijms252212391

**Published:** 2024-11-19

**Authors:** Maria A. Strelkova, Anna P. Tolstova, Vladimir A. Mitkevich, Irina Yu. Petrushanko, Alexander A. Makarov

**Affiliations:** Engelhardt Institute of Molecular Biology, Russian Academy of Sciences, 119991 Moscow, Russia; mitkevich@gmail.com (V.A.M.); irina-pva@mail.ru (I.Y.P.); aamakarov@eimb.ru (A.A.M.)

**Keywords:** Src kinase, SH4UD, REHT MD, protein structure, proto-oncogene, protein phosphorylation

## Abstract

Src kinase is one of the key regulators of cellular metabolism and is dysregulated in numerous diseases, including cancer, neurodegenerative diseases, and particularly Alzheimer’s disease. Despite its therapeutic importance, its full-length structure has never been obtained before, as it contains an intrinsically disordered regulatory region, SH4UD. The SH4UD region is crucial for Src activation, functional dimerization, and regulation by other kinases. In this study, we used the replica exchange molecular dynamics approach with a hybrid temperature and Hamiltonian tempering to obtain the conformational ensemble of full-length Src kinase in its non-phosphorylated state and in the presence of its two key regulatory phosphorylations: pY419 and pY530. The representative structures and simulation trajectories of non-phosphorylated pY419 and pY530 Src are available in open access. We demonstrate that pY419 phosphorylation, which is associated with Src activation, enhances its motility, whereas inhibited pY530 Src preserves relatively compact conformation. This study also provides insights into how SH4UD contributes to Src substrate binding, dimerization, and autophosphorylation, highlighting the putative role of 14-RRR-16 in this process.

## 1. Introduction

Src kinase is known to be the first well-described proto-oncogene and a key regulator of cellular metabolism [1]. The inhibitors of Src kinase are used as drugs in anti-cancer therapy [1]. Moreover, Src kinase targeting is also suggested as a potential therapeutic approach to the complex therapy of Alzheimer’s disease (AD) [2], which is nowadays the most widespread but incurable neurodegenerative disease. The complex nature of AD implies that it needs a complex multi-targeted therapeutic approach to be cured; still, the existing ways of treating AD lack multi-targeting [3,4].

Src kinase is known to be hyperactivated in AD. In turn, its hyperactivation is connected to glial dysfunction [5,6], impaired neuroplasticity [7], tau cleavage [8], neuroinflammation [9], pathological signaling [5], neurotransmitter regulation (i.e., NMDA receptors), hypersensitivity [10], and purinergic receptor signaling pathway mediation [11,12]), which are all disturbed or dysregulated in Alzheimer’s disease [13,14]. From this point of view, Src kinase is a prospective target for AD treatment. The investigation of its specific protein interactions and effective ways of inhibitor searching will contribute substantially to the treatment of diseases that are nowadays known as incurable.

The Src kinase domain composition is as follows (from N- to C-terminus) [1]: (a) SH4 domain (residues 2–19 for human Src); (b) Unique Lipid-Binding Domain (ULBD) (residues 60–67)—the region that is important for the lipid binding of Src kinase; (c) SH3 and SH2 regulatory domains (residues 84–145 and 151–248, respectively), which show high homology between different Src family kinases; and (d) SH1, or kinase, domain (residues 270–523), which is highly homologous between different tyrosine kinases. The SH4 domain and the ULBD are often united into regulatory SH4UD (in some papers, it is also called the Src N-terminal regulatory element, or SNRE [15]). Although a lot of effort has been put into obtaining the structure of Src, there is still no full-length model of the kinase, as SH4UD is intrinsically disordered and cannot be crystallized.

Despite its rather short length (83 amino acids), SH4UD contains up to 20 phosphorylation sites (according to PhosphoSitePlus [16]), which are proven to affect its functionality and correlate with Src hyperactivation in the pathology of various diseases. Still, understanding of the structural features of SH4UD, its interaction with the other domains, and the particular structural organization that mediates its impact on Src functionality remains poor. Few attempts were made to establish the structural patterns of the SH4UD domain of Src kinase experimentally or by modeling. In some papers, upon modeling approaches for intrinsically disordered proteins (IDPs), SH4UD of Src kinase was used as an example of IDP [17]. However, the behavior of such fragments strongly depends on their environment, meaning that SH4UD of Src kinase may act differently when being included in the whole molecule. The ability of SH4UD phosphorylations to regulate the Src activity and its effect on the folding of structured domains [18] implies the formation of direct contacts between the folded and unfolded parts of Src, which apparently determine the actual structural patterns of SH4UD.

Nowadays, Src kinase inhibitors work in a short range of specificity, targeting the highly conservative kinase domain and inhibiting other kinases. To design specific inhibitors and investigate the interaction interfaces between Src kinase and its protein partners, the unique domain that distinguishes Src from other SFKs, i.e., SH4UD, has to be taken into account [15]. Additionally, the role of SH4UD in the pathological activity of Src and its interaction with other proteins has also lately become increasingly discussed [19,20,21]. The absence of SH4UD in existing models of Src kinase may distort the results obtained with modeling, i.e., Src kinase docking to other molecules or inhibitor searching. In turn, the understanding of the SH4UD conformational ensemble will help describe specific Src kinase interactions and improve the effectiveness of Src-targeting therapy by developing highly specific inhibitors [15,22,23]. Thus, we obtained the structural ensemble of the full-length Src kinase using the REHT modeling dynamics approach. Additionally, we also obtained the structural ensembles of the key regulatory modifications of Src kinase that are known to crucially affect its structure and functionality: those phosphorylated by Y419 Src (activated state) and phosphorylated by Y530 (inhibited state). Both phosphorylations are assumed [24] to alter the Src kinase structure, either stimulating its motility (pY419) or reorganizing its domains in a compact inhibited globule (pY530) [24]. For Src kinase with Y419 phosphorylation, which is described to happen spontaneously in the absence of inhibitory modifications [1], for the first time, we obtained the structural model that includes structured domains and disordered SH4UD. This result allows us to confirm the assumption about the increased motility of autophosphorylated Src and suggest the role of SH4UD in the kinase activity of Src.

## 2. Results

### 2.1. Selection, Evaluation, and Verification of Starting Structures, MD Approaches, and Force Fields Applicable to Full-Length Src Kinase Modeling

**Figure 1:**
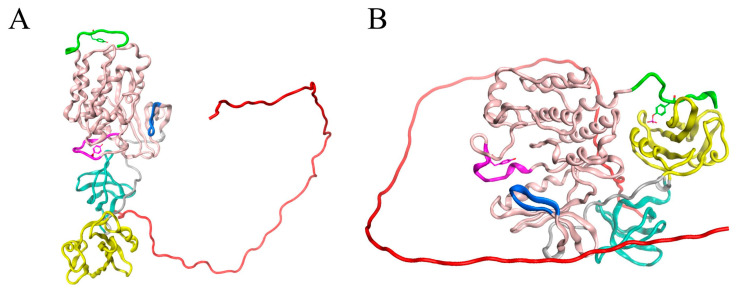
Starting structures for three Src kinase simulations. SH4UD is shown in red, SH3- in cyan, SH2- in yellow, kinase domain in pink ribbons. The activation loop containing Y419 is shown in purple, the C-terminus containing Y530 is shown in green, the ATP-binding site is shown in blue. (**A**) For non-phosphorylated Src kinase, the hybrid structure was constructed as follows: (a) residues 1–83 (SH4UD)—fully extended random coil from AlphaFold2; (b) residues 84–536—structured domains obtained from X-ray crystallography (PDB:1Y57). (**B**) Src kinase inhibitory phosphorylated at Y530 adopts a highly ordered structure with specific domain orientation, thus we used the X-ray crystallography results different from (**A**) to make a hybrid starting structure: (a) residues 1–83 (SH4UD)—fully extended random coil from AlphaFold2; (b) residues 84–536—structured domains obtained from AlphaFold2 as ensemble-averaged over 72 structures from RCSB PDB bank for P12931 entry.

Our first goal was to predict the structure of SH4UD using the artificial intelligence system AlphaFold2 [25]. As a result of AlphaFold2 prediction, we obtained the fully extended random coil conformation of SH4UD. Such random coil conformations are widely used as starting structures for the modeling of intrinsically disordered regions (IDRs) and intrinsically disordered proteins (IDPs) [26,27]; thus, we used SH4UD from AlphaFold2 to construct three hybrid starting structures. The coordinates for SH3-SH1 domains were taken from the RCSB PDB bank (PDB:1Y57 [28] for non-phosphorylated and pY419 Src and ensemble-averaged over 72 structures of pY530 Src kinase from the PDB bank, Figure 1).

The active phosphorylation of Src kinase at Y419 is not supposed to radically change the relative orientation of its structured domains as compared to non-phosphorylated Src [24]. Thereby, the hybrid starting structure for pY419 Src kinase modeling was constructed as follows: (a) residues 1–83 (SH4UD)—fully extended random coil from AlphaFold2; (b) residues 84–536—structured domains of non-phosphorylated Src kinase obtained from X-ray crystallography (PDB:1Y57) with additionally phosphorylated Y419.

The results of conventional MD can be easily distorted by the starting structure while the representative structures corresponding to distant local energy minima can never be achieved. Thus, in this study, we chose the replica exchange molecular dynamics approach, which has been shown to be appropriate for the modeling of IDPs and IDR-containing proteins [29]. Due to the large system size, we used hybrid replica exchange molecular dynamics with both temperature and Hamiltonian tempering (REHT) [30]. The modeling time of 200 ns per replica within 32 replicas was found to be sufficient to achieve the equilibrium conformations of the simulation according to convergence estimations. We chose the CHARMM36m force field as an optimal force field for modeling both intrinsically disordered and structured proteins [31].

### 2.2. Convergence of REHT MD Simulations

**Figure 2:**
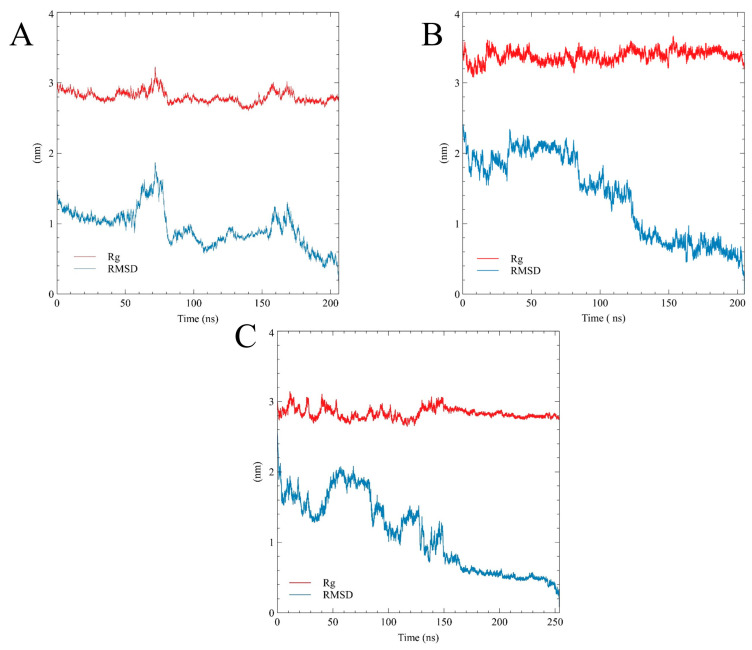
RMSD and gyration radii for non-phosphorylated Src (**A**), pY419 Src (**B**), and pY530 Src (**C**).

For the proteins that contain both structured and intrinsically disordered regions, defining the simulation time enough for the trajectories to achieve convergence is a tricky problem. In our study, simulations were performed for about 200 ns per replica, and the convergence time of each trajectory corresponded to the convergence time of its statistical quantities, i.e., root mean square deviation (RMSD) and gyration radius (Rg) distribution (Figure 2 and Figure S1). The resulting converged part of the trajectory was equal to 56–206 ns, 35–205 ns, and 53–253 ns for non-phosphorylated, pY419, and Y530 Src kinase simulations, respectively.

### 2.3. Conformational Flexibility of Src Kinase Depends on Its Key Regulatory Phosphorylations

**Figure 3:**
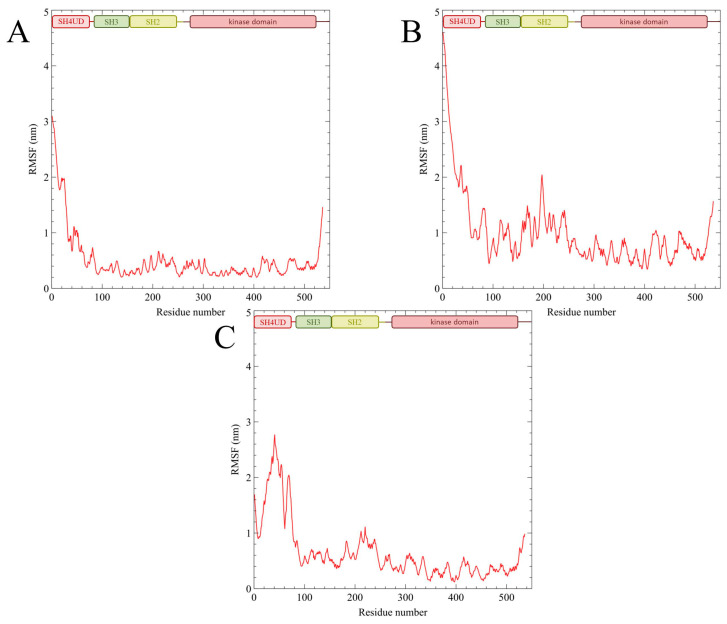
RMSF for non-phosphorylated Src (**A**), pY419 Src (**B**) and pY530 Src (**C**).

To evaluate the flexibility of the modeled proteins root mean square fluctuation (RMSF) was measured for the converged trajectories (Figure 3). The trajectories are available at Zenodo (https://www.authorea.com/doi/full/10.22541/au.172626192.29101974/v1, accessed on 13 September 2024).

During the simulation, structural fluctuations were mostly observed at SH4UD. The first 30–50 ns of the simulations reflected the conformational transition of SH4UD from an extended random coil to the globular-like conformation. Afterward, SH4UD untwisted again and refolded into a new, regulatory phosphorylation-specific conformational pattern. The RMSD graphs bounced (Figure 2), and the following trajectories demonstrated high convergence. Nevertheless, the flexibility of structured domains genuinely varied among the simulations. PY419 Src kinase (Figure 3B) demonstrated enhanced flexibility during the whole modeling, “plowing” the structured domains open for the first seconds of the simulation and remaining highly motile even after the transition. High motility of SH4UD is mostly provided at 83–132 ns of the modeling when it moves from the regulatory domains to the kinase domain, being nearly organized afterward. The peaky flexibility in this region, mostly pronounced for pY419 Src, is associated with the structured domain rearrangements, particularly with the linker between the regulatory and the kinase domains and their surroundings. Non-phosphorylated Src kinase (Figure 3A) underwent general compactization of the structured domains compared to the starting structure and retained a relatively rigid conformation with highly flexible SH4UD during the rest of the simulation. PY530 Src kinase (Figure 3C) is almost rigid, and its SH4UD also demonstrates increased orderedness, especially pronounced at its N-terminus and phosphorylated C-terminus.

### 2.4. The Src Kinase Regulatory Phosphorylations Alter Its Domain Organization

**Figure 4:**
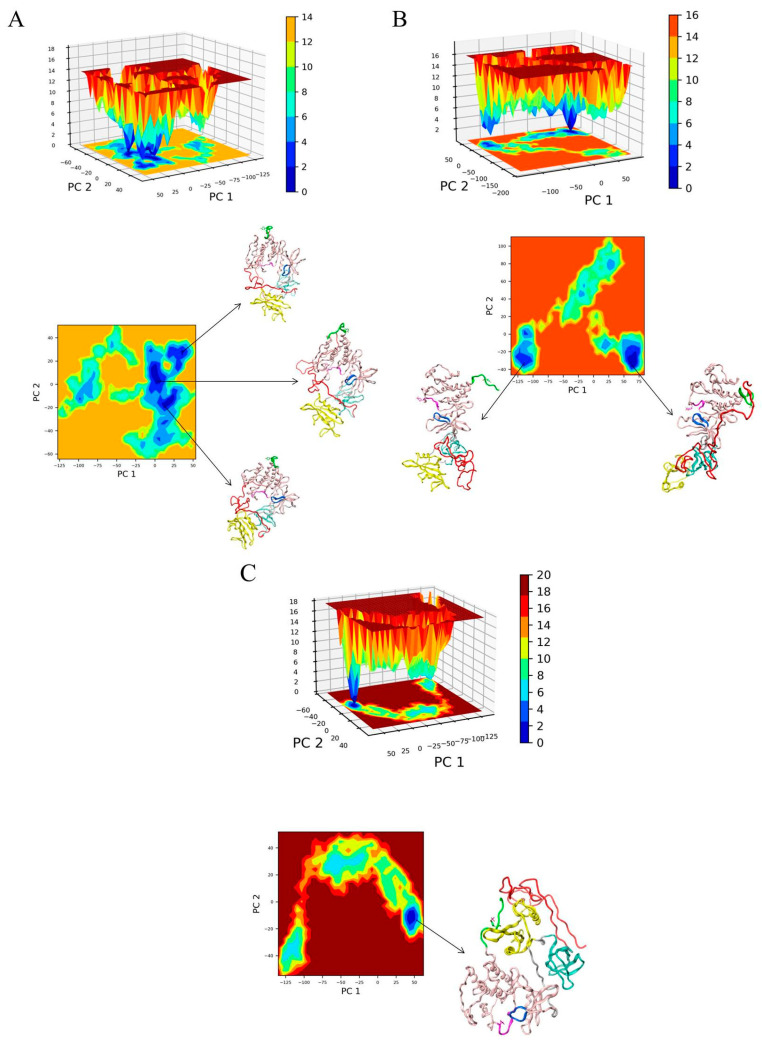
Free energy landscapes for the converged trajectories of non-phosphorylated Src (**A**), pY419 Src (**B**), and pY530 Src (**C**) obtained using principal component analysis. PC 1- principal component 1; PC 2–principal component 2. The scale bar is drawn in kcal/mol. SH4UD is shown in red, SH3- in cyan, SH2- in yellow, kinase domain in pink ribbons. The activation loop containing Y419 is shown in purple, the C-terminus containing Y530 is shown in green, the ATP-binding site is shown in blue.

To sample the conformational dynamics of Src kinase, we calculated Gibbs free energy landscapes (FELs) from the converged parts of the trajectories for non-phosphorylated Src, pY419 Src and pY530 Src. Principal component analysis was performed for the converged part of the trajectories. FELs were obtained as a function of the first and the second principal components (Figure 4).

**Figure 5:**
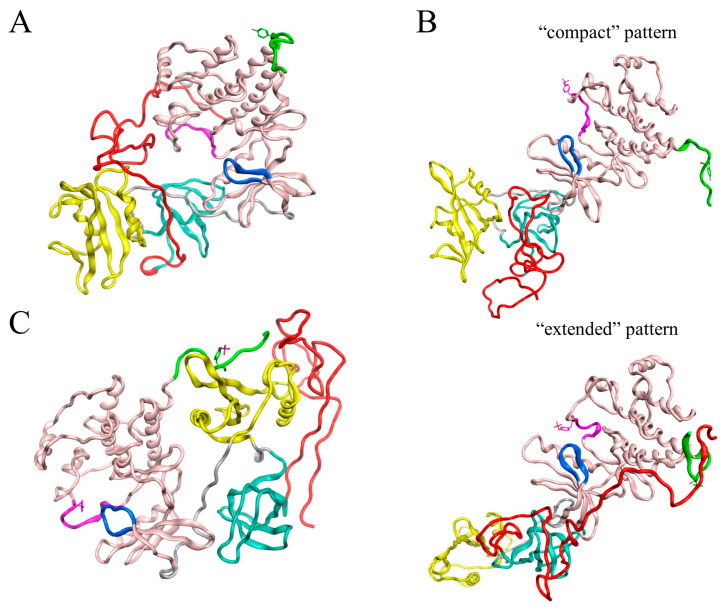
The representative conformations for non-phosphorylated Src (**A**), pY419 Src (**B**) and pY530 Src (**C**) obtained using principal component analysis as first cluster centroids. For pY419, representative conformations from two local energy minima are provided. SH4UD is shown in red, SH3- in cyan, SH2- in yellow, kinase domain in pink ribbons. The activation loop containing Y419 is shown in purple, the C-terminus containing Y530 is shown in green, the ATP-binding site is shown in blue.

For each trajectory, all conformations corresponding to zero Gibbs energy were extracted. They were clustered using the DBSCAN algorithm. Centroid structures for the first five clusters represent the ensemble of energetically favorable conformations that a single Src kinase can adopt in solution (Figure 5). The PDB files are available at Zenodo (https://www.authorea.com/doi/full/10.22541/au.172626192.29101974/v1, accessed on 13 September 2024).

The representative conformation for non-phosphorylated Src kinase (Figure 5A) reflects that in this state Src kinase comprises a relatively close, or compact, structure. SH4UD remains interacting with the regulatory domains and the kinase domain. Meanwhile, starting from the same conformation as the non-phosphorylated one, the structured domains of pY419 Src kinase (Figure 5B) finally form an open conformation. In this state, SH3-, SH2- and kinase domains are arranged in a row, and two distinct conformational patterns of SH4UD are observed, named hereafter as “compact” (37–83 ns of the simulation) and “extended” (132–205 ns of the simulation). The “compact” pattern is mostly represented by the random coil that interacts with the SH3-domain. During the simulation, SH4UD undergoes a conformational transition to the “extended” pattern, displacing along the structured domains and forming a noticeable beta sheet with the C-terminus. PY530 Src (Figure 5C) reflects negligible alterations at the structured domains compared to the starting structure, and its SH4UD fluctuates faintly near SH2- and SH3-domains.

### 2.5. The Structured Domains of Src Kinase Interact with SH4UD Differently in the Presence of Key Regulatory Phosphorylations

**Figure 6:**
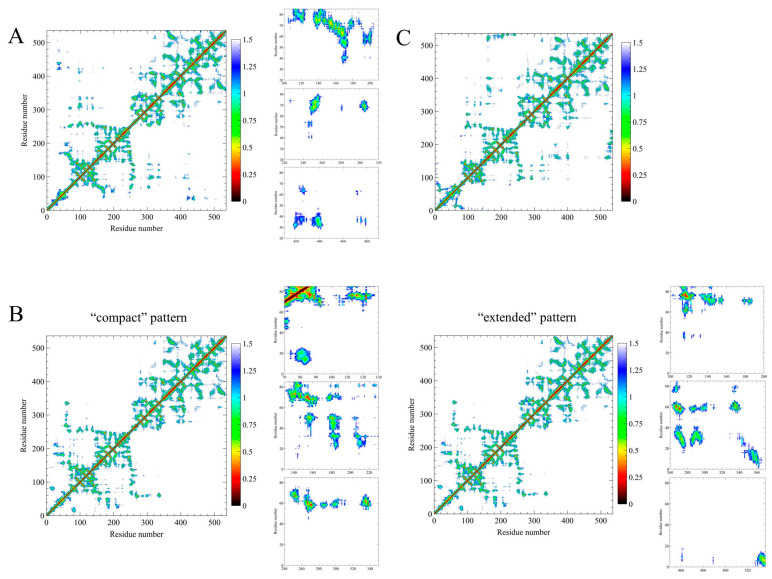
The pairwise residue contacts between SH4UD (residues 1–83) and structured domains (residues 84–536) for non-phosphorylated Src (**A**), “compact” and “extended” patterns of pY419 Src (**B**), and pY530 Src (**C**). The scale bar is drawn in nm.

To identify the key interactions between SH4UD and the structured part of Src kinase, we constructed contact maps averaged over the converged parts of the trajectories. For each converged trajectory, the average distances between each residue pair were calculated (Figure 6).

The pairwise residue contacts between SH4UD and structured domains are substantially different between the converged parts of the simulations. In non-phosphorylated Src kinase (Figure 6A), SH4UD mainly interacts with SH3-domain. The contacts are formed by residues 60–74 of SH4UD (Figure S2), whereas its N-terminal part is mostly exposed to the solution. We also observe the direct interaction between SH4UD and the activation loop that contains the Y419 phosphorylation site (residues 415–420). For pY419 Src kinase (Figure 6B), SH4UD showed the highest contact rate with the structured domains. In the “compact” pattern, it forms only minor contacts with the kinase domain. The N-terminus of SH4UD (residues 2–19) does not interact with structured domains. The “extended” pattern reflects the stable, mostly unalternative contact formation between the N-terminus of SH4UD and the kinase domain. The contacts are also formed with the unphosphorylated C-terminus that contains Y530. In both patterns, region 60–74 (Figure S2) interacts with SH3-, SH2- and kinase domains. For pY530 Src kinase (Figure 6C), negligible contacts of SH4UD with structured domains are observed.

### 2.6. The Src Kinase Regulatory Phosphorylations Determine the Structural Patterns of SH4UD

**Figure 7:**
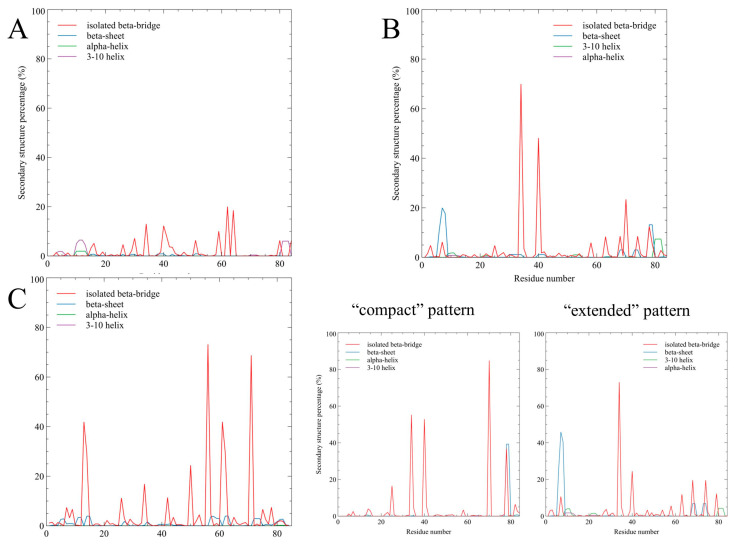
The secondary structure population for SH4UD (residues 1–83) for non-phosphorylated Src (**A**), “compact” and “extended” patterns of pY419 Src (**B**), and pY530 Src (**C**).

To investigate the structural patterns of SH4UD within different regulatory phosphorylations of Src kinase, we analyzed the secondary structure populations of SH4UD residues. Secondary structure assignment for the three converged REMD trajectories was made using the DSSP [32] approach and VMD 1.9.3 [33] software (Figure 7).

The fraction of secondary structures for SH4UD is sufficiently different between the converged parts of the simulations. In non-phosphorylated Src kinase (Figure 7A), the secondary structures of SH4UD are sparsely populated yet diverse. The isolated beta-bridge between residues 34 and 40 is observed in approximately 10% of the converged simulation (Figure S3). Other isolated beta-bridges are formed by residues 59, 62, 64 (about 20% of the converged simulation). Residues 10–14 and 81–82 form 3-turn and alpha helices for approximately 7–10% of the converged simulation (Figure S4). Other secondary structure elements are mostly presented by beta structures and are much less populated. For pY419 Src kinase (Figure 7B), secondary structures are mostly formed within the “extended” pattern of SH4UD. The isolated beta-bridge formed by residues 34 and 40 was populated for more than 20% of the pattern simulation time (Figure S3), and for residue 34, the isolated beta-bridge population exceeds 70%. Beta structures are also formed by residues 3–9 and 63–79 (up to 45% and 20% of the pattern simulation time, respectively, Figure S5). The helical content is almost negligible and is mostly observed in residues 10–12 and 80–82 (Figure S4). In the “compact” pattern, the isolated beta bridge between residues 34 and 40 is conserved for more than 50% of the simulation (Figure S3). A distinct beta bridge is also formed between residues 70 and 78. In pY530 Src kinase (Figure 7C), secondary structures of SH4UD are mostly represented by isolated beta bridges that are never observed in other simulations, i.e., residues 56–71 (Figure S6), 14–61, 26–42, implying the general tendency of the pY530 Src kinase structure towards the increased self-orderedness.

## 3. Discussion

Src kinase contains three structured domains that are conservative among all Src family kinases (SFKs): SH3- and SH2-regulatory domains and a kinase domain. Additionally, Src kinase has a regulatory N-terminal SH4 domain and a unique domain, collectively termed SH4UD, which determine its specific protein–protein interactions. The structure of the conservative SH3-, SH2- and kinase domains is well described. According to Src kinase structures obtained by X-ray crystallography [28,34], when Src kinase is phosphorylated at Y530, regulatory domains flip over the kinase domain and are stabilized in a specific position due to the interaction with the phosphorylated C-terminus and the SH2-domain [24]. In this state, the entire peptide adopts a compact inhibited conformation [34]. Dephosphorylation of Y530 leads to relaxation of the molecule and restitution of the domain orientation [24]. When the kinase domain is released and returns to its functional state, it undergoes autophosphorylation at Y419. The structural rearrangements associated with Src autophosphorylation are still unknown. However, it is generally assumed that autophosphorylation enhances the overall flexibility of the protein, increasing its ability to catch the substrate and enhancing its enzyme activity [24].

**Figure 8:**
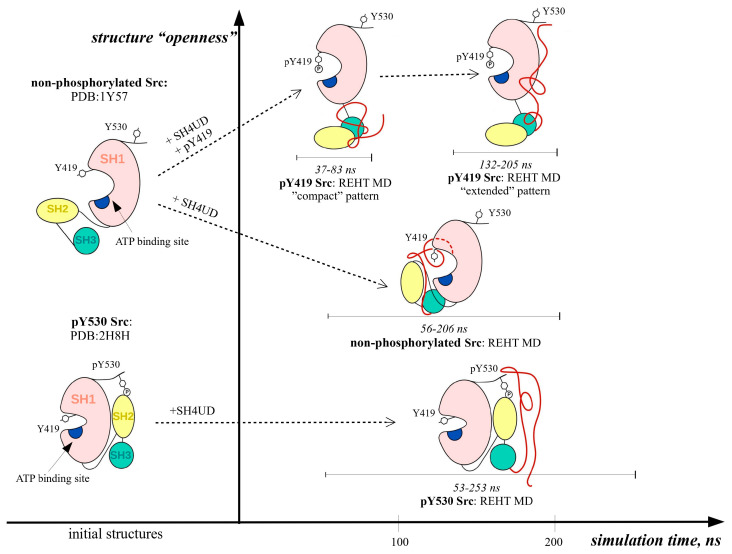
The graphical representation of MD predicted full-length Src domain rearrangement in the presence of key regulatory phosphorylations. Structure “openness” is presented in a schematic way, which corresponds to the acknowledged concepts of inhibited and open forms of Src [24] and correlates with the estimations of average Rgyr for each conformation. SH4UD is shown in red, SH3- in cyan, SH2- in yellow, kinase domain (SH1) in pink. The ATP-binding site is shown in blue.

REHT MD approach allows us to overcome the energy barriers between different energetically favorable conformations and obtain relevant structures from the sterically distant starting ones for a shorter simulation time [30]. The representative structures obtained in our modeling demonstrate that the overall composition and the domain orientation of the ordered part of the molecule do not change significantly during the simulation compared to the starting ones based on the results of the X-ray crystallography [28,34] (Figure 8).

For non-phosphorylated Src (Figure 5A), the structured domains get closer to each other, forming a more compact structure than obtained by X-ray crystallography; yet, the internal structure of the domains, as well as their relative orientation, remains intact. This indicates that our results confirm the previous ones obtained experimentally. Nevertheless, we cannot entirely exclude the impact of the starting structure, which is the limitation of all current modeling approaches for large systems.

As previously assumed by other research groups [24,35], phosphorylation of Src kinase at Y419 (Figure 5B), did not induce the crucial domain rearrangements compared to non-phosphorylated Src, except for the increased openness and flexibility of the structured part of the protein (Figure 3B). This versatile behavior may be associated with the enhanced enzyme activity of autophosphorylated Src kinase. On the contrary, the compactly ordered structured domains of pY530 Src kinase remained almost intact during the simulation (Figure 5C) and demonstrated overall rigidity (Figure 3C). The absence of any conformational rearrangements reaffirms the X-ray crystallography data on which the starting structure of our modeling was based.

**Figure 9:**
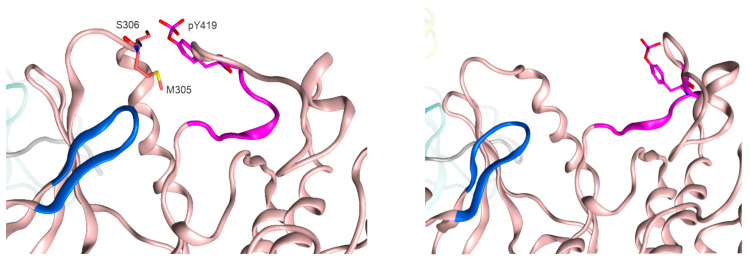
The interaction between pY419 and residues 305–309 and free pY419 of Src kinase. SH3- is shown in cyan, SH2- in yellow, kinase domain in pink ribbons. The activation loop containing Y419 is shown in purple, the ATP-binding site is shown in blue.

Additionally, we observed an intriguing interaction between pY419 and residues 305–309 (Figure 9) of the kinase domain. Although we find it difficult to suggest the certain physiological function of this interaction, it may contribute to the general motility of the kinase domain, which is demanded for effective ATP and substrate binding [24]. These features of the domain organization of Src phosphorylations may induce the specific contact formation and consequent rearrangements of SH4UD that we have observed during modeling.

Regarding SH4UD itself, some attempts have been made to describe its conformational diversity as an independent peptide using NMR and molecular dynamics approaches. In [17], the authors used the REHT MD approach for the 94-amino acid N-terminal fragment. They predicted three regions with significant propensity of helicity, one of which was also observed in our simulations (residues 10–13). The other two helices were predicted at residues 42–45 and 69–71, which, according to our analysis, interact with the structured domains and form beta bridges. The comparison between the results obtained for the SH4UD peptide [17] and for the whole Src kinase (Figure 7) demonstrates the substantial impact of the structured domains and their phosphorylations on the structural pattern of SH4UD. This particularly means that the results obtained for the intrinsically disordered fragments of the peptides cannot be extrapolated when this fragment is considered as a part of a whole protein.

There are some structural elements formed independently in our simulations that have never been observed before. This is, in particular, the enhanced helicity at residues 10–14 and 80–82 (Figure 7 and Figure S4) and the impressive propensity of the isolated beta bridge between residues 34 and 40 (Figure 7 and Figure S3) for non-phosphorylated and pY419 Src. Some of these secondary structure elements may presumably play a regulative role, altering the accessibility of the regulatory phosphorylation sites S12 [36] and T37 [37] (for which this regulatory mechanism was previously suggested [17]), which are known to be phosphorylated by protein kinase C and protein kinase A, respectively. T37 is highly conservative among different species and SFKs [21], indicating the regulatory importance of this site. pS12 and pT37 Src are known to be associated with Src kinase hyperactivation and are found in cancer cells [37,38]. We presume that the phosphorylation sites S12 and T37 are regulatory elements independent of Src phosphorylation at Y419, as their structural environment (i.e., helices at 10–13 and beta bridge 34–40) remains unaltered for both non-phosphorylated and pY419 Src simulations.

Previously, NMR analysis [39] revealed that the SH3 domain plays a prominent role in the conformational organization of SH4UD, which is in good accordance with our results. Still, the discrepancies in the exact residues involved in the interaction can be explained by the absence of the other structured domains in the NMR experiment. Additionally, the residual dipolar coupling (RDS) analysis [40] showed that SH4UD is partially structured at residues 60–64 and 67–74 when included in the whole protein. We demonstrated the stable interaction of these regions with SH2-, SH3- and kinase domains observed for non-phosphorylated and pY419 Src kinase (Figure 6 and Figure S2) and found various secondary structure elements that genuinely stabilize these regions in pY530 Src kinase (Figure 7 and Figure S6). These interactions strictly fix residues 60–74 in certain configurations, strongly decreasing their mobility. This is supported by the pronounced minima of RMSF values for this region (Figure 3). Thus, we conclude that our modeling is in proper agreement with the experimental data obtained earlier by an independent research group.

While the helicity of residues 10–14 has been previously detected in SH4UD peptide modeling [17], the beta bridge between residues 34 and 40 has never been detected before and obviously requires the presence of other Src domains. What is more, this bridge is located in a mainly unstructured region. Such bridges may hardly be detected using existing experimental approaches. These include RDS, which is unsuitable for demonstrating the spot interactions between IDR fragments, and NMR, which has been used in previous studies mostly to detect the prolonged patterns or spot interactions that are altered under different conditions. While there is still no methodology for the experimental detection of spot interactions, modeling of full-length proteins obviously remains the only way to predict them.

The REHT MD results revealed that the conformational behavior of SH4UD is determined not only by the presence of structured domains of Src but also by the specific changes associated with their phosphorylations (Figure 8). SH4UD shows different conformational patterns among three simulations (i.e., non-phosphorylated, pY419 and pY530 Src). Here, we highlight the key features of each of them. First, the “compact” and “extended” patterns (Figure 5) reflect a unique configurational behavior of SH4UD in pY419 Src. At the secondary structure level, the beta sheets at residues 68–69 and 73–74 and the isolated beta bridge between residues 63–79 in pY419 Src form a unique secondary structure environment for the phosphorylation sites S69 [36], S70 [41] and S75 [36] (Figure 7B), which are hyperphosphorylated in different cancer types. The accessibility of these phosphorylation sites thus depends on the presence of pY419, in a counterweight to the S12 and T37 sites. At the same time, SH4UD of pY530 Src is characterized by distinctive orderedness, as it is shown by the RMSF analysis (Figure 3C). It is conditioned by a wide variety of highly populated beta bridges that are never observed in two other simulations (Figure 7C). All these observations demonstrate the pivotal role of Src key phosphorylations in the formation of secondary structure and conformational pattern of SH4UD.

**Figure 10:**
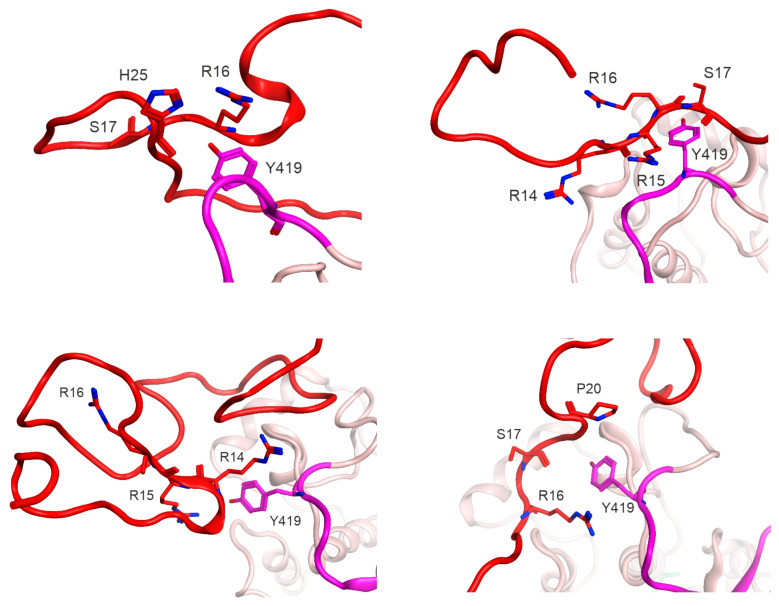
The interaction between Y419 and SH4UD in non-phosphorylated Src kinase. SH4UD is shown in red, kinase domain in pink ribbons. The activation loop containing Y419 is shown in purple. The atoms of the interacting residues are shown in ribbon colors, with nitrogen atoms highlighted in blue.

There is a lot of experimental data on the influence of SH4UD on the Src activity and protein partner binding, yet the exact mechanisms still remain unknown. Numerous phosphorylations of SH4UD are noticed to correlate with the activation level of Src kinase [15,42]. Further, the autophosphorylation of Src kinase at Y419 has been shown to be impaired in the absence of SH4UD [43]. Here, we for the first time propose the first step in the phosphorylation of Src kinase. In the simulation of non-phosphorylated Src, we observed a stable interaction between the unphosphorylated Y419 and residues 14–19 of SH4UD that wrap around it (Figure 10). This region is mainly composed of positively charged arginines (14-RRR-16), which form a favorable environment for ATP orientation and its consequent hydrolysis. This observation may shed light on the mystery of the autophosphorylation at the Y419 site, which is spatially distant from the ATP binding site and whose mechanism remains unknown. In addition, this region contains a regulatory S17 phosphorylation site [36]. This site is known to be hyperphosphorylated in numerous cancer types, and its mutation S17A leads to a drastic decrease in Y419 phosphorylation levels [19]. We assert the possible phosphate transition from S17 to Y419 as a putative mechanism of Src hyperactivation in the presence of S17 phosphorylation that has been observed experimentally by another research group [19].

SH4UD is also proposed to play a central role in the functional dimer formation of Src, the process that is crucial for its signaling activity [43]. Dimerization requires Src autophosphorylation [43]; thus, we speculated on how the specific conformational patterns of SH4UD of pY419 Src may mediate dimerization. Phosphorylation at Y419 enhances the secondary structure formation at residues 3–9 (Figure 7) and the interaction between the N-terminal region and the non-phosphorylated C-terminus (Figure S5). This region, particularly K5, K7 and K9, has been previously described to be crucial for Src dimerization [44]. The beta structure formation between region 3–9 and the C-terminus observed in our modeling possibly mediates the way in which Src autophosphorylation promotes its dimerization, as it may analogously bind the C-terminus of another Src molecule. In all, further investigation is needed to predict the dimerization mechanism based on the corresponding intermolecular interaction, as well as the mechanisms underlying the impact of the SH4UD phosphorylations [19] on this process.

There is an assumption [35] that the phosphate from pY419 may be transposed intramolecularly to Y530. We tend to claim that the direct transition is sterically impossible due to the spatial distance between the phosphorylation sites. Still, we assert that SH4UD may somehow mediate this process, switching between the “compact” and “extended” states and possibly transferring the phosphate from the phosphorylated A-loop to the non-phosphorylated C-terminus of pY419 Src kinase.

*Limitations.* Among all the MD approaches, the REMD approach allows us to obtain conformational ensembles instead of a single equilibrium structure and simultaneously saves computational power in the most effective way. However, the increasing orderedness and instability of the modeled system that contains both intrinsically disordered and structured fragments may impede replica exchange. This effect was especially pronounced for the simulation of highly ordered pY530 Src kinase. The conformational ensembles obtained from simulation appear as numerous alternative conformations that are inconvenient to use. For all simulations, we treated first cluster centroids calculated for all conformations corresponding to free energy landscape minima as representative conformations of Src kinase. The representative conformations do not reflect the conformational diversity of the protein; however, they show the main tendencies and conformational features of partially ordered proteins. At the same time, the diversity of secondary structures and intramolecular contacts remains thoroughly described with the statistical analysis (DSSP and contact maps) of the converged trajectories. Our results are in agreement with experimental data and can be used to shed some light upon the role of SH4UD in Src kinase functionality and the effect of the autophosphorylation on Src kinase structural organization.

## 4. Materials and Methods

### 4.1. Structure Preparation

The structure used as the initial model for modeling the Src kinase in the inhibited state was taken from the AlphaFold2 program database [25]. The structure of SH1-SH3 domains of this conformation corresponds to the superposition of 72 conformations of the inhibited Src kinase from the PDB bank (e.g., 1.1 Å RMSD with PDB:2SRC [34], resolution 1.50 Å, and 0.8 Å RMSD with PDB:2H8H [45], resolution 2.20 Å, both crystallized without SH4UD). The SH4UD domain forms an unstructured loop that wraps around the protein in a wide arc and is exposed along its entire length in solution (Figure 1). Coordinates for residues 534–536 were generated manually. The coordinates for structured domains of non-phosphorylated Src kinase were taken from X-ray crystallography (PDB:1Y57). For the SH4UD domain, the coordinates were taken from AlphaFold2 calculated for inhibited Src kinase. SH4UD was manually added to the 1Y57 pdb file to reproduce the same arrangement relative to the structured part of the protein as in pY530 Src kinase.

Since the structure of Src kinase in the pY419 active state is unknown, we used the same structure as for the active unphosphorylated state but manually phosphorylated at Y419.

Each system consisted of Src kinase protein, 150 mM of NaCl (which corresponds to the physiological concentration) and 75,000 water molecules for compact pY530 Src or 100 000 water molecules for open (pY419 and non-phosphorylated Src kinase) states, enough to avoid interaction with the periodic replicas.

### 4.2. MD Simulation Parameters

In this study, the parallel-tempering REHT method was used for the simulations utilizing the modeling protocols described in [26]. We chose the REHT method since SH4UD is intrinsically disordered and together with its activity-associated motility makes this protein poorly suited for classical modeling. Additionally, full-length Src kinase consists of approximately 8000 atoms, which significantly slows down classical temperature REMD calculations. The choice of a hybrid temperature and Hamiltonian tempering allowed us to strike a balance between adequate modeling time and high quality of the results. We used 32 replicas resulting in a total simulation time of 6.5 to 8.1 microseconds. A temperature interval of 300–330K was used for water and ions and 300–380 K for the protein atoms. A replica exchange was attempted every 1500 steps. Exchange probabilities for non-phosphorylated, pY419 and pY530 Src kinase were 14.8%, 15.5% and 14.8%, respectively. Simulation times were 206, 205 and 253 ns, respectively, in the time range used elsewhere [26,27]. The replica index distribution is provided in Figure S7.

In all simulations, Charmm36m force field [31] with TIP3P water model was used. All simulations were carried out with the GROMACS 2022.3 package [46]. The modelings were conducted in an NPT ensemble with V-rescale temperature coupling and C-rescale pressure coupling, particle mesh Ewald technique for electrostatics, 1.2 nm cut-offs for Coulomb and van der Waals potentials, 2-fs time step and periodic boundary conditions.

### 4.3. Convergence Estimations

The convergence of simulations was assessed by comparing the Rg distributions at several time intervals (Figure S1) and by the time evolution of Rg and RMSD values (Figure 2). The replica index distribution over simulation (Figure S7) shows that the first replica visited all windows during simulation. It indicates that during the modeling process, Src kinase successfully explored the full range of conditions set up for the simulation.

Yet we performed the simulation long enough to observe the convergence, we cannot guarantee that we obtained all the possible conformations of SH4UD during our modeling. Due to the complexity of the system, the temperature range and simulation time were relatively short (80 K and 205 ns). Still, we used a large water simulation box to avoid the interaction between the periodic replicas, the physiological concentrations of salt, and the force field suitable for both structured and disordered proteins to perform our simulation in the most precise way.

### 4.4. Statistical Analysis

For the analysis, we used only the converged part of the trajectory, which was obtained as described in Section 2.2. For the time evolution of the structural features, the following GROMACS [46] utilities were used: *gmx rms*, *gmx gyrate* and *gmx rmsf*. Averaged contact maps were calculated using *gmx mdmat* command. The DSSP algorithm implemented in VMD 1.9.3 software [33] was used to calculate the secondary structure distribution over time.

Free energy surfaces (FESs) were calculated using the GROMACS [46] *gmx sham* program. For each trajectory, all conformations corresponding to zero Gibbs energy on the FES were extracted using *gmx trjconv* command. They were subjected to structural alignment and clusterization using the DBSCAN algorithm and Biomol2Clust v 1.2 software (https://biokinet.belozersky.msu.ru/Biomol2Clust, accessed on 10 February 2024).

## 5. Conclusions

In this study, for the first time, we managed to obtain the complete full-length conformational ensemble of Src kinase in its non-phosphorylated state and in the presence of two key regulatory phosphorylations: pY419 and pY530. Non-phosphorylated Src kinase demonstrated more compact conformation compared to the starting structure, and its local energy minima differed mostly by the SH4UD configuration. The structured domains of pY419 Src shift to a widely open conformation during the simulation, showing enhanced motility. In this state, SH4UD comprises two alternative conformations, either locating near regulatory domains (“compact”) or being extended along the kinase domain (“extended”). Phosphorylation at Y530, in turn, enhances the rigidity of the protein and, particularly, SH4UD. The key regulatory phosphorylations of Src kinase strongly affect the secondary structure propensity of SH4UD, determining the accessibility of the SH4UD phosphorylation sites, which are associated with different diseases and metabolic impairment. Our study suggests some clues on how SH4UD contributes to the Src kinase autophosphorylation, as well as substrate binding and dimerization. The structures available on Zenodo (https://www.authorea.com/doi/full/10.22541/au.172626192.29101974/v1, accessed on 13 September 2024) are the first full-length Src structure that can be usable for bioinformatics needs, such as protein docking and searching for specific Src kinase ligands affine to its unique domain. This massive computational work will help researchers in cancer, Alzheimer’s disease or other diseases involving Src kinase dysregulation to push the horizons of the existing therapeutic paradigms.

## Data Availability

Standardized ensembles of structures for non-phosphorylated, pY419 and pY530 Src kinase are available directly at Zenodo (https://www.authorea.com/doi/full/10.22541/au.172626192.29101974/v1, accessed on 13 September 2024).

## Supplementary Materials

The supporting information can be downloaded at: https://www.mdpi.com/article/10.3390/ijms252212391/s1.
