# Supplementary material for: Structure of Full-Length Src Kinase and Its Key Phosphorylated States: Molecular Dynamics Study
Source: Int J Mol Sci. 2024 Nov 19;25(22):12391. doi: 10.3390/ijms252212391 (PMC11594451; doi:10.3390/ijms252212391)
Supplement: Supplementary file 1 [file ijms-25-12391-s001.zip › ijms-3263032-supplementary.pdf]

Supplementary Materials for article "MD Predicted Structure of Full-length Src Kinase and Its Key Phosphorylated States" by Maria A. Strelkova, Anna P. Tolstova, Vladimir A. Mitkevich, Irina Yu. Petrushanko and Alexander A. Makarov

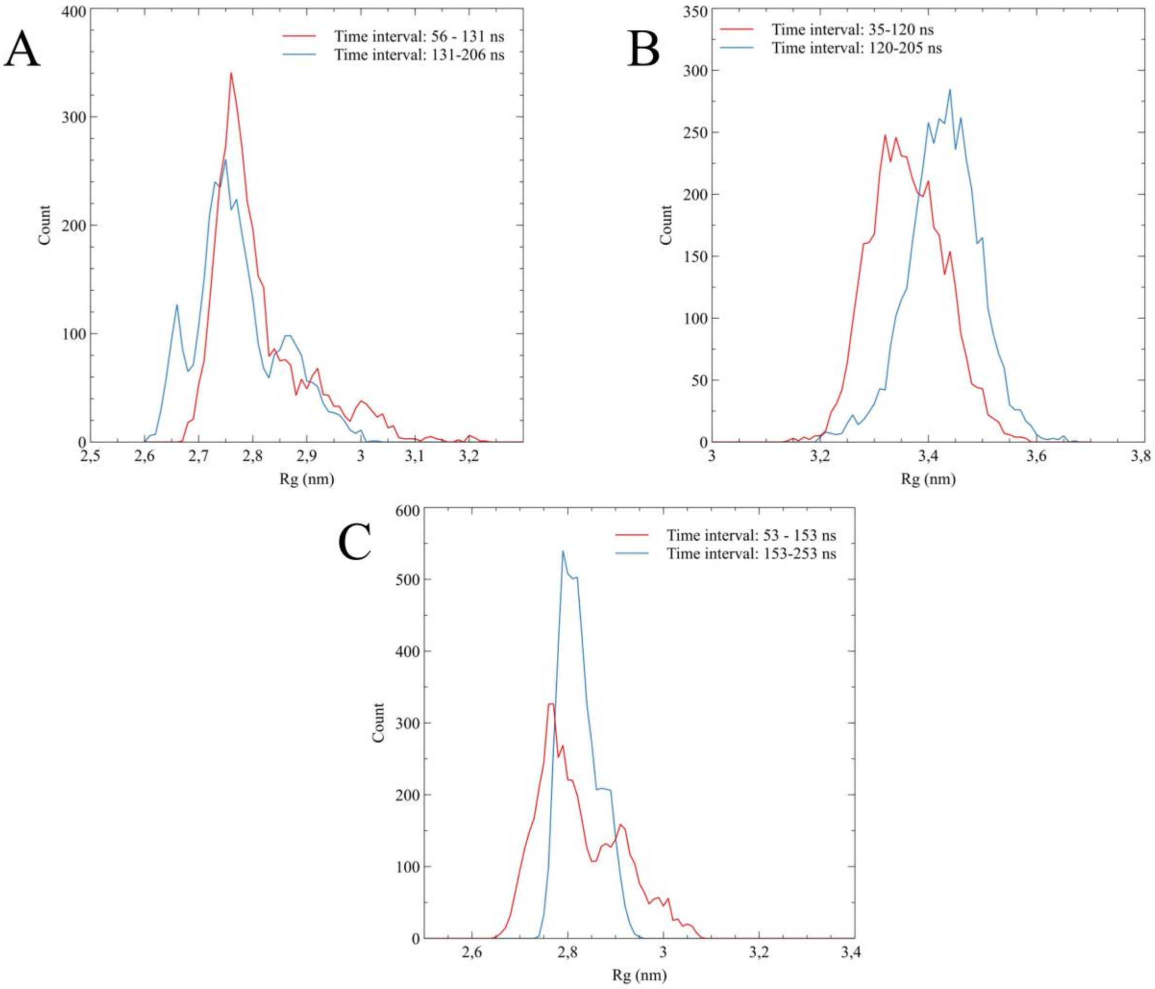

Figure S1. Gyration radius distribution for non-phosphorylated Src (A), pY419 Src (B) and pY530 Src (C).

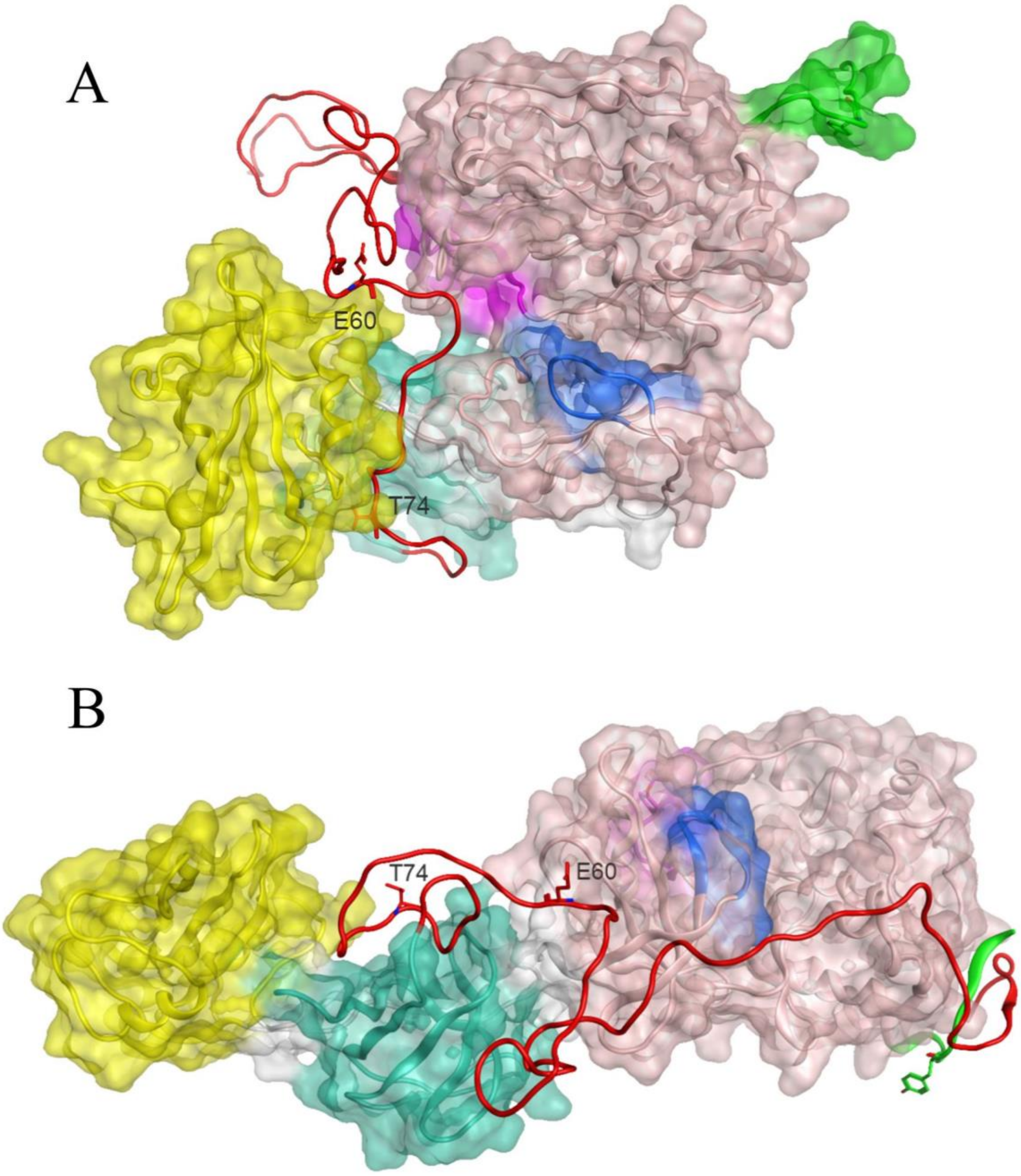

Figure S2. Partially ordered conformations of residues 60-74 illustrated by the example of non-phosphorylated Src (A) and the “extended” pattern of pY419 Src (B). SH4UD is shown in red ribbons, SH3- in green, SH2- in yellow, kinase domain in pink surfaces. The activation loop containing Y419 is shown in purple, the C-terminus containing Y530 is shown in green, the ATP-binding site is shown in blue.

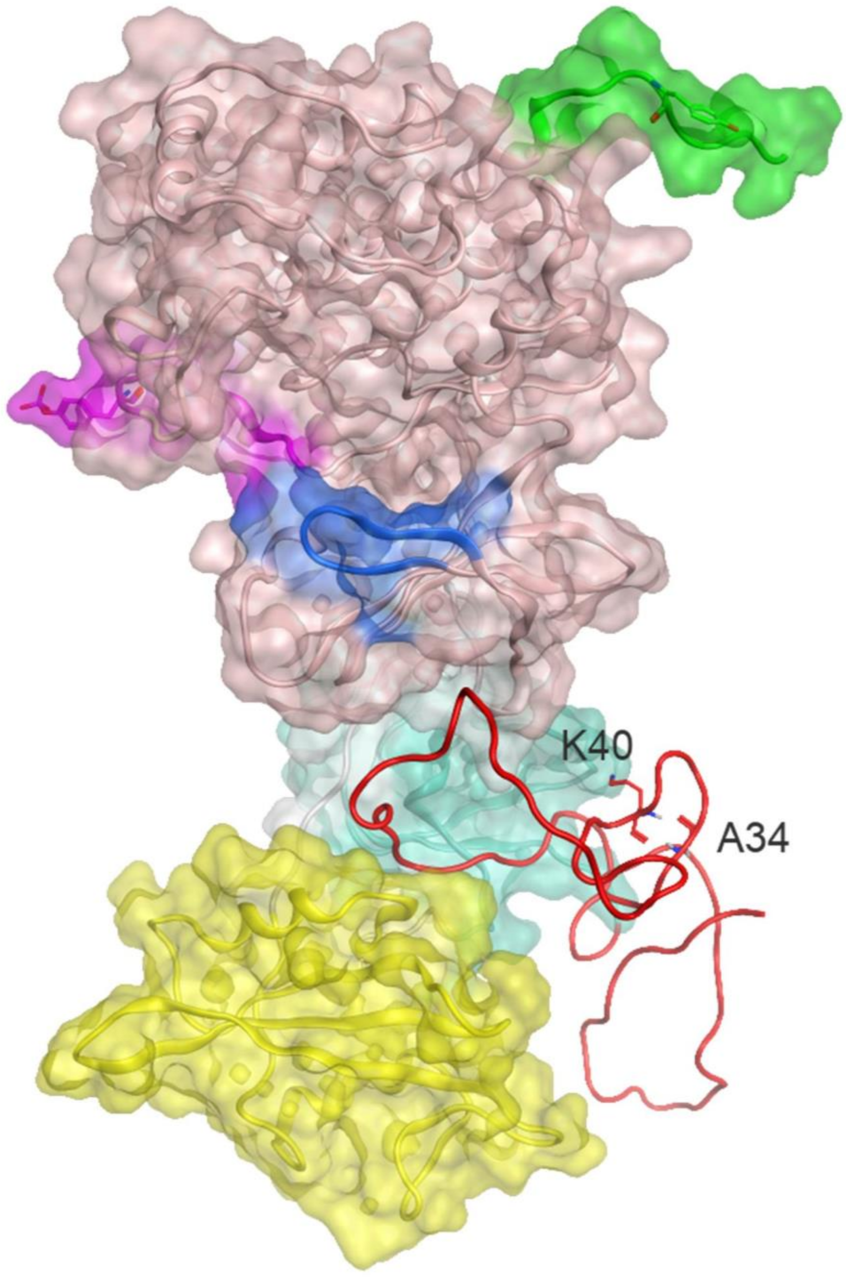

Figure S3. An isolated beta-bridge between residues 34 and 40 illustrated by the example of the "extended" pattern of pY419 Src kinase. SH4UD is shown in red ribbons, SH3- in green, SH2- in yellow, kinase domain in pink surfaces. The activation loop containing Y419 is shown in purple, the C-terminus containing Y530 is shown in green, the ATP-binding site is shown in blue.

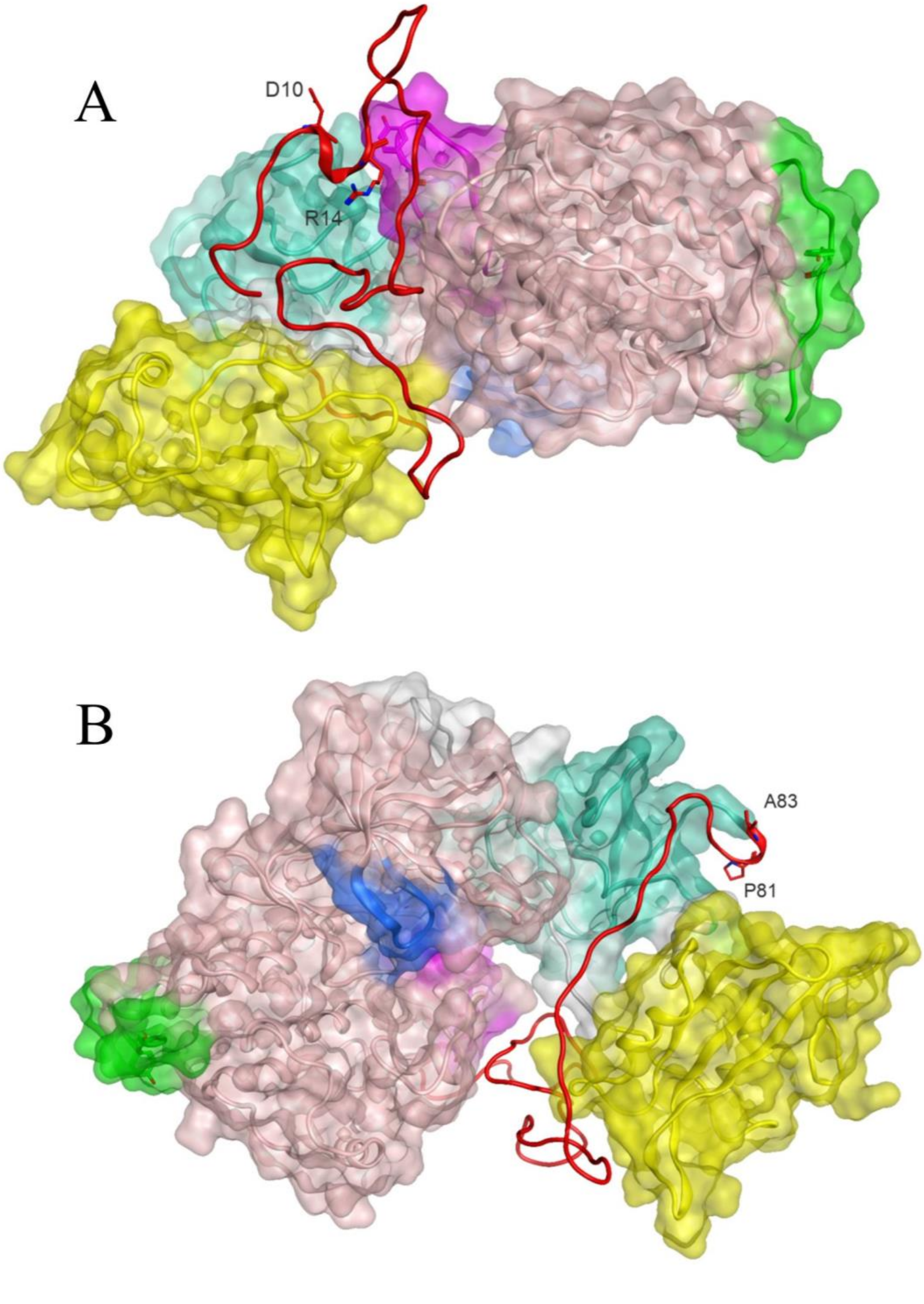

Figure S4. Helicity of residues 10-14 (A) and 81-83 (B) illustrated by the example of non-phosphorylated Src. SH4UD is shown in red ribbons, SH3- in green, SH2- in yellow, kinase domain in pink surfaces. The activation loop containing Y419 is shown in purple, the C-terminus containing Y530 is shown in green, the ATP-binding site is shown in blue.

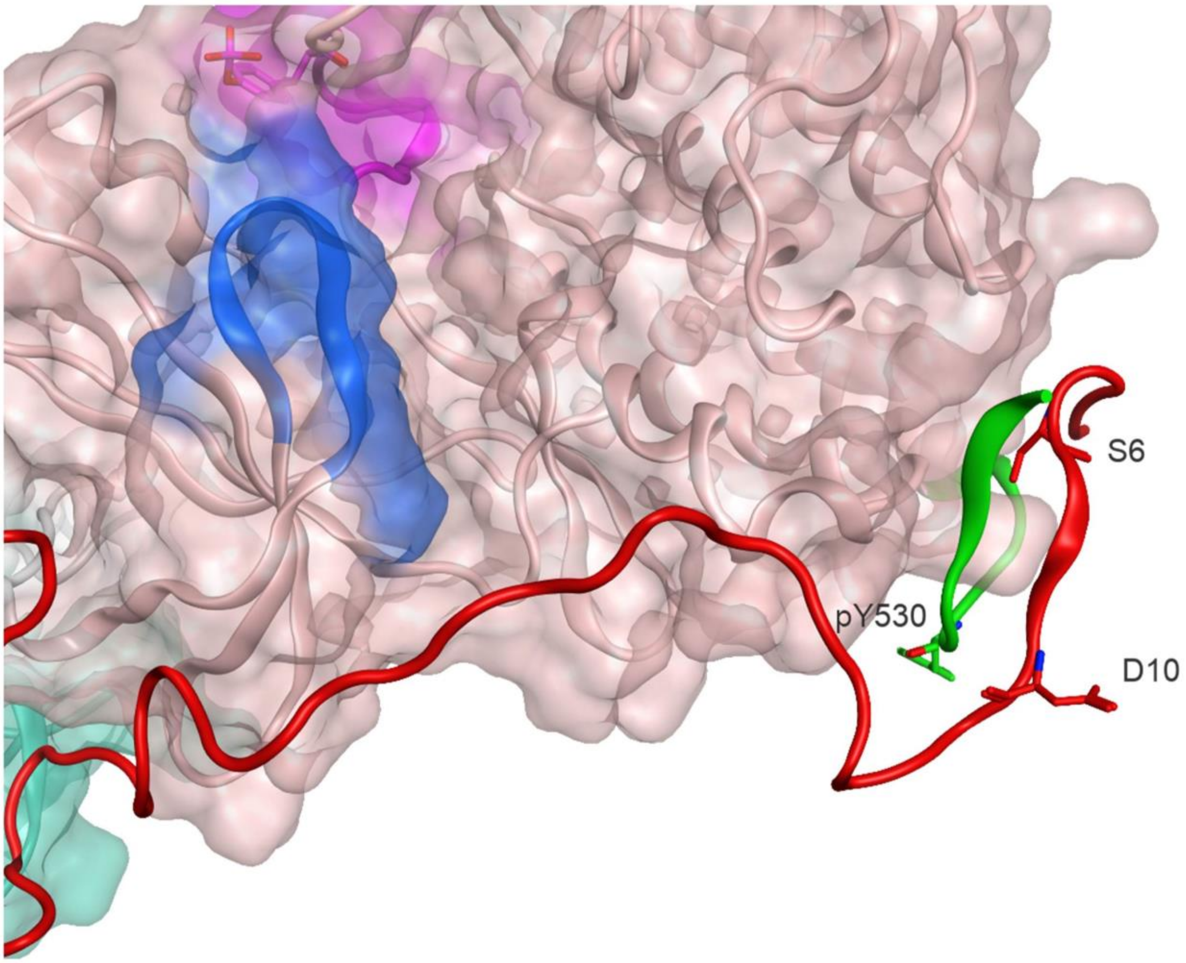

Figure S5. The beta-sheet between SH4UD and the C-terminus for pY419 Src kinase. SH4UD is shown in red ribbons, SH3- in green, SH2- in yellow, kinase domain in pink surfaces. The activation loop containing Y419 is shown in purple, the C-terminus containing Y530 is shown in green, the ATP-binding site is shown in blue.

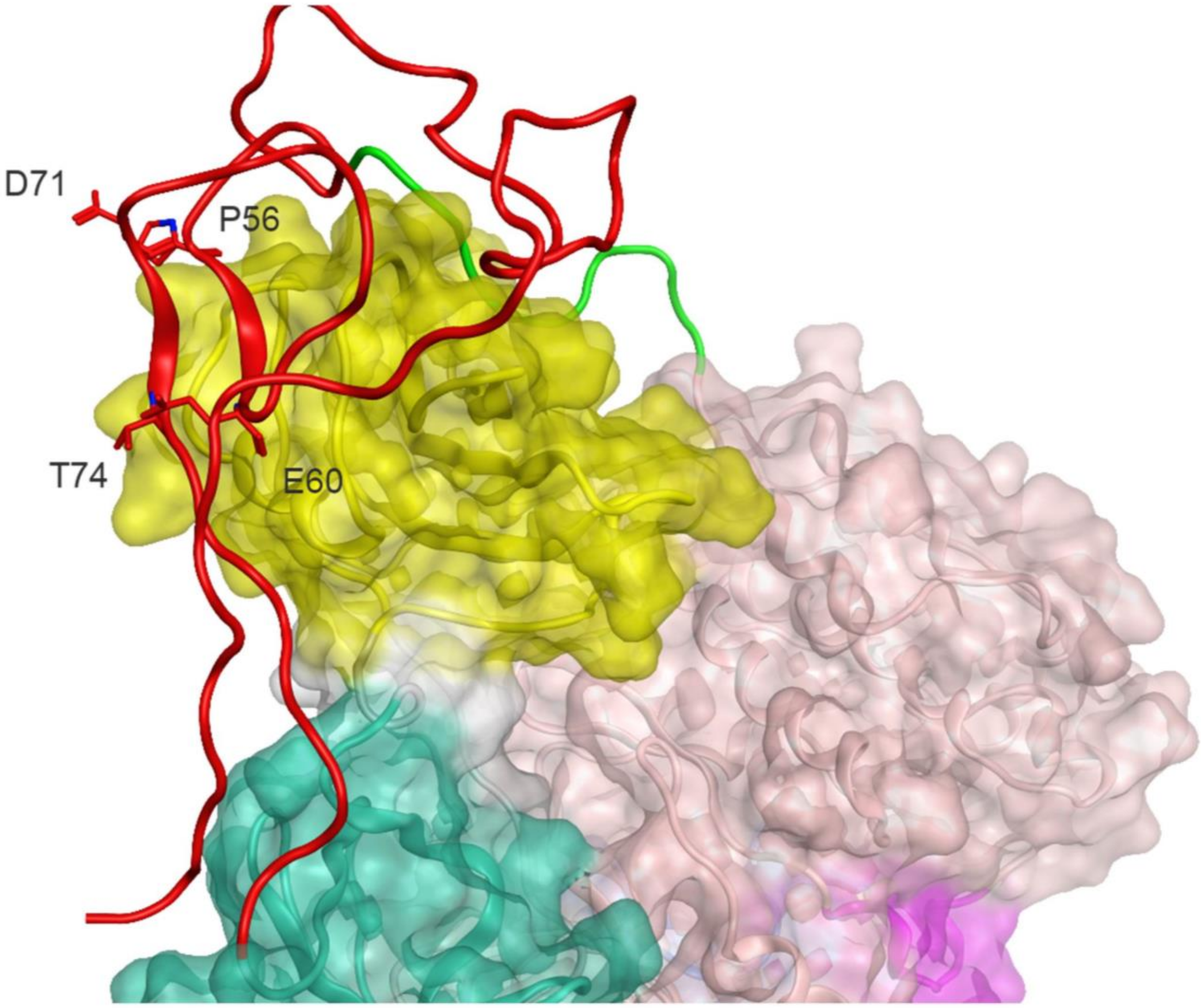

Figure S6. Beta-sheet formation between regions 56-60 and 71-74 for pY530 Src. SH4UD is shown in red ribbons, SH3- in green, SH2- in yellow, kinase domain in pink surfaces. The activation loop containing Y419 is shown in purple, the C-terminus containing Y530 is shown in green, the ATP-binding site is shown in blue.

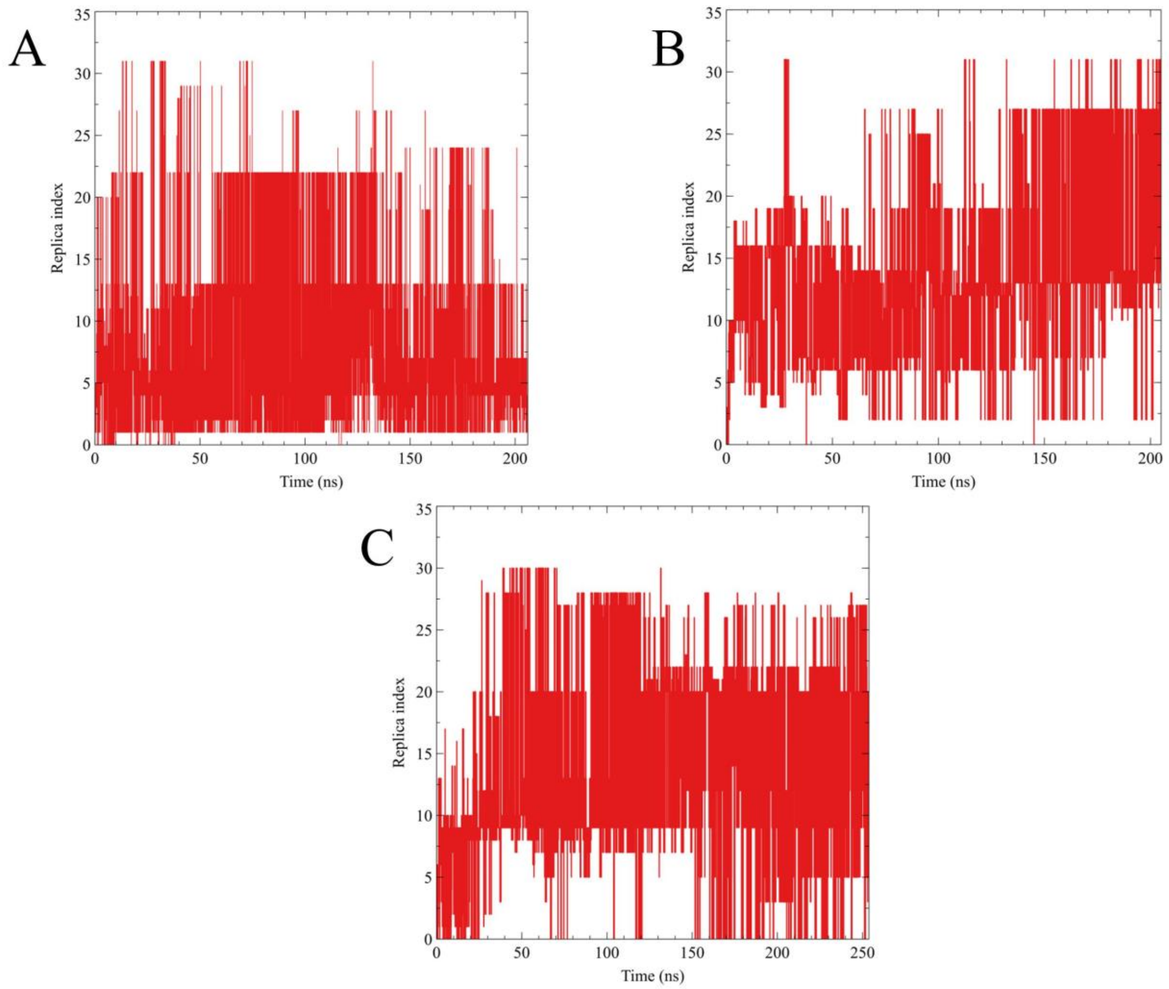

Figure S7. Replica index distribution for the first replica during the REMD simulation for non-phosphorylated Src (A), pY419 Src (B) and pY530 Src (C).
